# Prognostic significance of preoperative neutrophil-to-lymphocyte ratio in surgically resected schwannomas

**DOI:** 10.3389/fonc.2023.1099384

**Published:** 2023-02-10

**Authors:** Kento Takahara, Ryota Tamura, Yuki Kuranari, Kosuke Karatsu, Takenori Akiyama, Masahiro Toda

**Affiliations:** ^1^ Department of Neurosurgery, Keio University School of Medicine, Tokyo, Japan; ^2^ Department of Neurosurgery, Kawasaki Municipal Hospital, Kawasaki-ku, Kanagawa, Japan

**Keywords:** schwannoma, neutrophil-to-lymphocyte ratio, neurofibromatosis type 2, prognostic factor, retreatment

## Abstract

**Objective:**

The goal of schwannoma resection is to control the tumor while preserving neurological function. Schwannomas have a variable postoperative growth pattern, therefore preoperative prediction of a schwannoma’s growth pattern is favorable. This study aimed to examine the relationship between preoperative neutrophil-to-lymphocyte ratio (NLR) and postoperative recurrence and retreatment in patients with schwannoma.

**Methods:**

We retrospectively examined 124 patients who underwent schwannoma resection in our institution. Associations between preoperative NLR, other patient and tumor characteristics, and tumor recurrence and retreatment were analyzed.

**Results:**

Median follow-up was 2569.5 days. Postoperative recurrence occurred in 37 patients. Recurrence that required retreatment occurred in 22. Treatment-free survival (TFS) was significantly shorter in patients with NLR ≥2.21 (*P* = 0.0010). Multivariate Cox proportional hazards regression showed that NLR and neurofibromatosis type 2 were independent predictors of retreatment (*P* = 0.0423 and 0.0043, respectively). TFS was significantly shorter in patients with NLR ≥2.21 in the following subgroups: sporadic schwannoma, primary schwannoma, schwannoma ≥30 mm in size, subtotal resection, vestibular schwannoma, and postoperative recurrence.

**Conclusions:**

Preoperative NLR ≥2.21 before surgery was significantly associated with retreatment after schwannoma resection. NLR may be a novel predictor of retreatment and assist surgeons in preoperative surgical decision making.

## Introduction

Schwannomas are benign tumors that originate from Schwann cells of the cranial and peripheral nerves, and have a variable growth pattern (1). Clinical outcome after their surgical resection is related to extent of removal (2–6), which in the future could be assisted by new imaging modalities besides classic magnetic resonance imaging (MRI) (7). The goal of schwannoma resection is to control the tumor while preserving neurological function (8–11). Accurate preoperative prediction of a schwannoma’s growth pattern might assist surgeons with clinical decision making regarding aggressiveness of resection and need for adjuvant radiotherapy. Ki-67 is a commonly used proliferative marker for several types of tumors; however, it cannot be evaluated before surgery and its significance in schwannoma is controversial (3, 5).

Inflammation promotes tumor development throughout all stages of tumorigenesis (12). Systemic inflammation and immune system activation is broadly reflected by the neutrophil-to-lymphocyte ratio (NLR), an inexpensive, easily measured, and readily available blood test. A high NLR has been associated with worse overall survival in many solid malignant tumors (13–17). A relationship between NLR and refractory intracranial benign tumor has also been demonstrated in other studies (18–22). In a previous study, we showed that preoperative NLR ≥2.6 was significantly associated with shorter progression-free survival in all grades of meningioma, including World Health Organization grade I (22). This study aimed to examine the relationship between preoperative NLR and postoperative recurrence and retreatment in patients with schwannoma.

## Methods

### Study design and clinical data

We retrospectively reviewed 270 patients who underwent surgical schwannoma resection in our institution from February 2010 to February 2018. The study received institutional review board approval (reference number, 20050002) and all patients provided written informed consent. Patients who received steroids or immunosuppressive drugs before preoperative laboratory testing, those with systemic infection, and with a history of malignancy were excluded. We also excluded those with incomplete clinical, laboratory, or radiological data.

The following data were obtained from the medical records: age at time of surgery, sex, neurofibromatosis 2 (NF2) status, tumor origin, primary/recurrent tumor, solid/cystic tumor, brain compression, neurological symptoms, and extent of removal. Tumor origin was determined using gadolinium-enhanced T1-weighted MRI. Extent of removal was determined using MRI after surgery.

MRI was performed every 6 to 12 months after surgery. In patients who underwent gross total resection (GTR), tumor recurrence was defined as the appearance of new tumor at the surgical site. In patients who underwent subtotal resection (STR), recurrence was defined as residual tumor growth ≥2 mm. Recurrence-free survival (RFS) was defined as the time from the date of surgery to the date of tumor recurrence or last imaging follow-up. Because not all schwannoma recurrences require treatment, treatment-free survival (TFS), a relatively new measure of disease control (23), was also evaluated. TFS was defined as the time from the date of surgery to the date of retreatment decision or last follow-up.

### Laboratory data

Absolute neutrophil, lymphocyte, monocyte, and platelet counts and concentrations of albumin, C-reactive protein, and fibrinogen were routinely obtained before surgery and the following inflammatory parameters calculated (24–29): NLR, lymphocyte-to-monocyte ratio (LMR), platelet-to-lymphocyte ratio (PLR), and prognostic nutritional index (PNI). PNI was calculated using the following formula: (10 × albumin concentration) + (0.005 × lymphocyte count).

### Statistical analysis

Statistical analyses were performed using JMP 16 software (SAS Institute, Cary, NC, USA). Continuous variables are expressed as means with standard deviation and were compared using the Mann–Whitney U test. Categorical variables are expressed as numbers with percentage and were compared using Fisher’s exact test. RFS and TFS were estimated using the Kaplan–Meier method and compared using the log-rank test. Univariate and multivariate Cox proportional hazards regression were used to evaluate the influence of variables on RFS and TFS. Receiver operating characteristic (ROC) curves were constructed to determine optimal cut-off values for each variable. *P <*0.05 was considered significant.

## Results

### Patient characteristics and laboratory data

**Figure 1:**
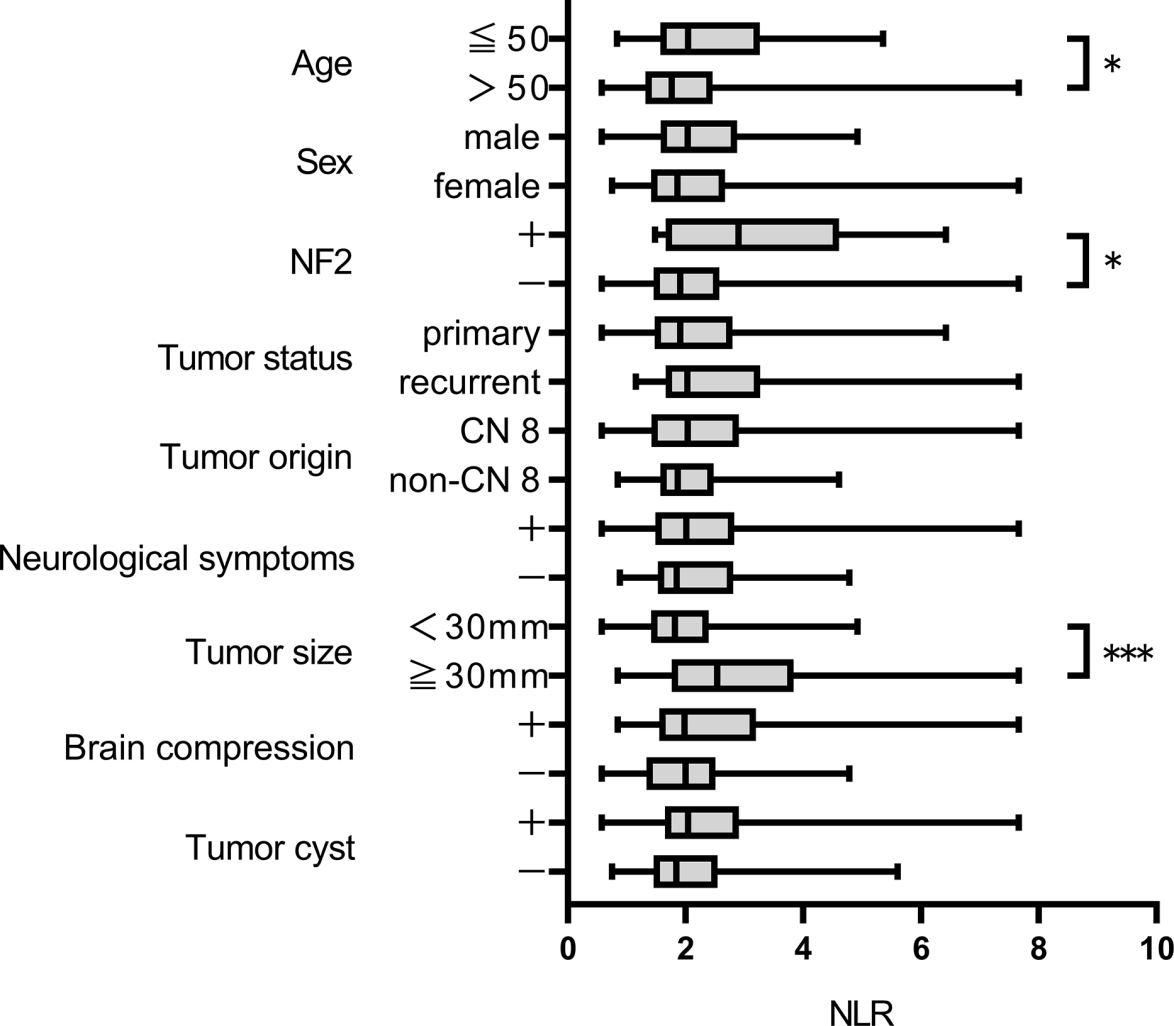
NLR value according to clinical characteristics *p < 0.05, ***p < 0.001.

**Figure 2:**
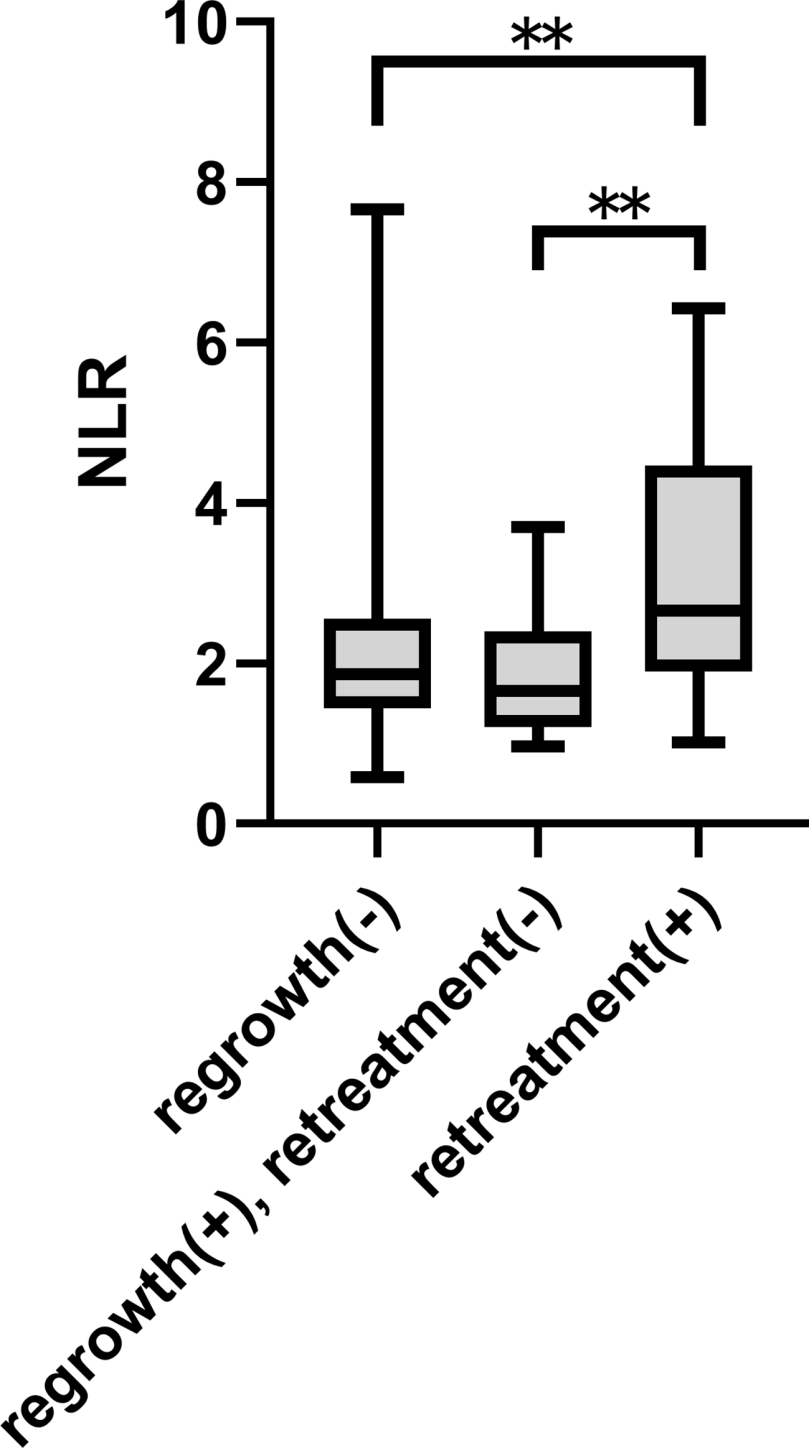
NLR value according to postoperative outcome **p < 0.01.

After excluding 146 patients based on criteria, 124 patients were included for analysis. Median follow-up was 2569.5 days. Postoperative recurrence occurred in 37 patients; recurrence that required retreatment occurred in 22. ROC curves were constructed for each variable using two outcomes, recurrence and retreatment. The optimal NLR cut-off value for recurrence and retreatment was 2.03 and 2.21, respectively. The area under the curve for recurrence and retreatment was 0.6039 and 0.7273, respectively (**Supplementary Figure 1**). **Table 1** and **Supplementary Table 1** show patient and tumor characteristics overall and with patients stratified by NLR cut-off value. The stratified groups were similar except for extent of resection. As shown in **Figure 1**, NLR value was significantly higher in younger patients and those with NF2 and tumor size ≥30 mm. NLR value was significantly higher in the retreatment group than the no recurrence and the recurrence but no retreatment groups. This suggests that NLR might be an important predictor of retreatment. NLR did not significantly differ between the no recurrence and the recurrence but no retreatment groups (**Figure 2**).

**Table 1:** Patient and tumor characteristics.

Clinical feature	All cases	Baseline NLR		P value
		<2.21	≧2.21	
		No.(%)	No.(%)	
Patient number	124	75 (60.5)	49 (39.5)	
Age (means ± SD)		49.8 ± 12.9	47.0 ± 14.8	0.2694
Sex				0.2689
Male	55	30 (54.5)	25 (45.5)	
Female	69	45 (65.2)	24 (34.8)	
NF2				0.1104
+	11	4 (36.4)	7 (63.6)	
-	113	71 (62.8)	49 (37.2)	
Tumor status				>0.9999
primary	109	66 (60.6)	43 (39.4)	
recurrent	15	9 (60.0)	6 (40.0)	
Tumor origin				0.2212
CN 8	91	52 (57.1)	39 (42.8)	
non CN 8	33	23 (69.7)	10 (30.3)	
Neurological symptoms				0.5629
+	110	65 (59.1)	45 (40.9)	
-	14	10 (71.4)	4 (28.6)	
Tumor size (mm)		24.8 ± 13.0	29.1 ± 12.0	0.0713
Brain compression				0.3416
+	79	45 (57.0)	34 (43.0)	
-	45	30 (66.7)	15 (33.3)	
Tumor cyst				0.3610
+	59	33 (55.9)	26 (44.1)	
-	65	42 (64.6)	23 (35.4)	
Removal rate				**0.0228**
GTR	46	34 (73.9)	12 (26.1)	
non-GTR	78	41 (52.6)	37 (47.4)	

NLR, neutrophil-to-lymphocyte ratio; SD, standard deviation; NF2, neurofibromatosis type 2; CN, cranial nerve; GTR, gross total resection. Factors which made statistically significant differences are shown in bold.

### Survival analysis

**Figure 3:**
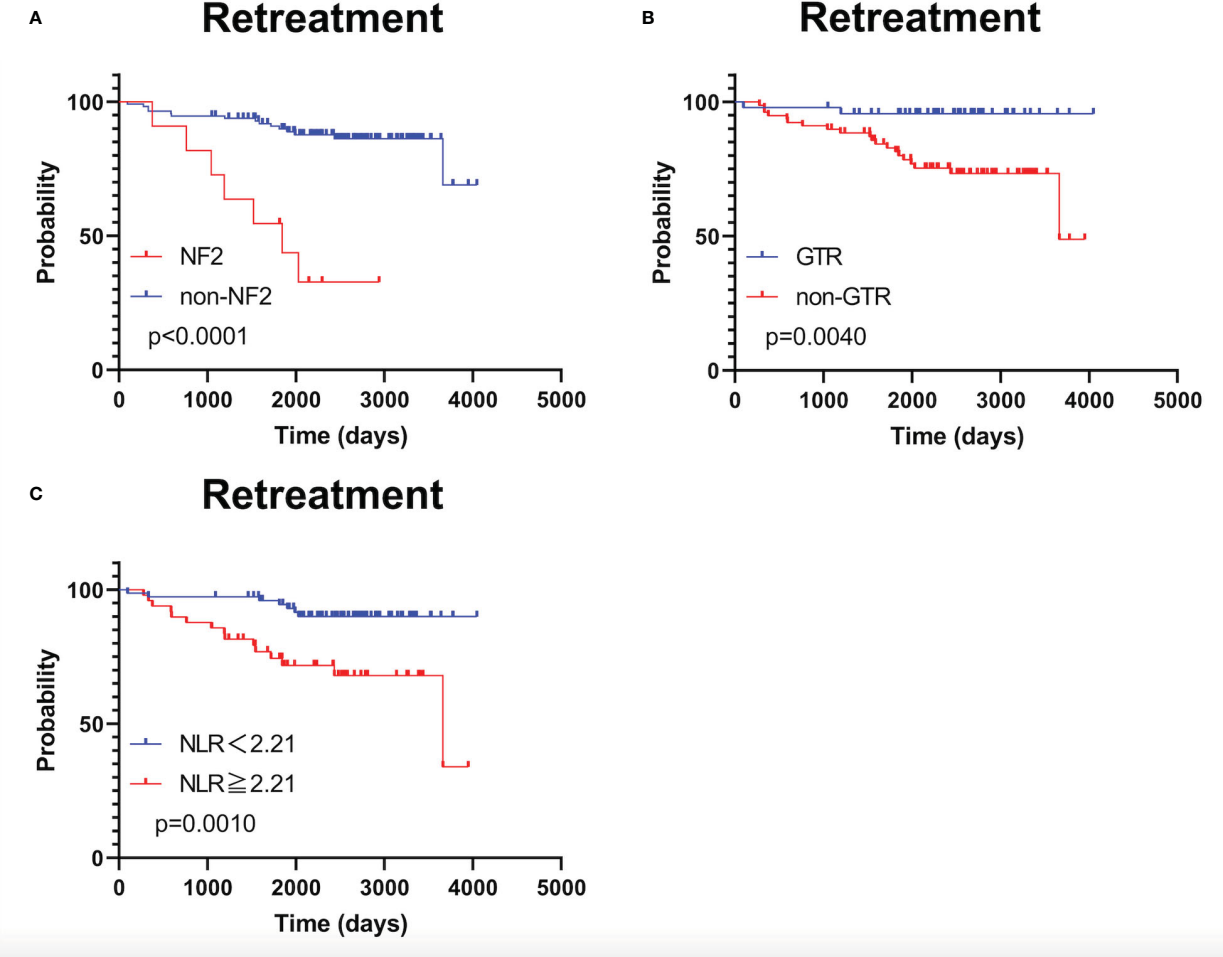
Kaplan–Meier treatment-free survival curves with patients stratified according to neurofibromatosis type 2 status **(A)**, extent of tumor removal **(B)**, and optimal neutrophil-to-lymphocyte ratio cut-off value **(C)**.

As shown in **Supplementary Figure 2**, RFS was significantly shorter in patients with NF2 than in patients without NF2 (*p <*0.0001); RFS did not significantly differ between patients stratified by NLR using a cut-off value of 2.03 (*p =* 0.088). TFS was significantly shorter in patients with NF2 and in those who underwent STR (*p <*0.0001 and p = 0.0040, respectively; **Figure 3A, B**). TFS was significantly shorter in patients with NLR ≥2.21 (*p* = 0.0010; **Figure 3C**).

### Univariate and multivariate analyses

Because NLR was associated with TFS but not RFS, univariate and multivariate Cox proportional hazards regression was performed to investigate the influence of variables on TFS (**Tables 2**, **3**). Although LMR was also associated with shorter TFS, NLR had a higher statistical power than LMR. The multivariate analysis showed that NF2 and NLR ≥2.21 were independent predictors of retreatment (*P* = 0.0043 and 0.0423, respectively).

**Table 2:** Univariate Cox proportional hazards regression analysis.

Variables	HR	95% CI	p value
Age (>50)	0.60	0.25-1.49	0.2719
Sex (male)	0.86	0.37-2.01	0.7271
**NF2 (+)**	**7.02**	**2.80-17.60**	**<0.0001**
Tumor status (recurrent)	1.66	0.56-4.92	0.3598
Origin (CN 8)	0.77	0.31-1.90	0.5707
Neurological symptoms (+)	0.57	0.19-1.69	0.3082
**Tumor size (≧30mm)**	**3.08**	**1.31-7.25**	**0.0098**
Brain compression (+)	1.96	0.72-5.33	0.1863
**Cyst (+)**	**2.49**	**1.01-6.12**	**0.0467**
**Removal rate (non-GTR)**	**6.40**	**1.50-27.40**	**0.0123**
PLT (>227000/μL)	0.51	0.22-1.19	0.1196
CRP (>0.025 mg/dL)	0.74	0.27-2.05	0.5657
Fibrinogen (>274mg/dL)	1.97	0.62-6.21	0.2495
**NLR (≧2.21)**	**4.07**	**1.65-10.02**	**0.0023**
**LMR (<4.11)**	**3.29**	**1.39-7.75**	**0.0065**
PLR (≧116.2)	2.51	0.84-7.45	0.0988
PNI (≧52.7)	1.64	0.60-4.43	0.3304

NF2, neurofibromatosis type 2; CN, cranial nerve; GTR, gross total resection; WBC, white blood cell; PLT, platelet; CRP, C-reactive protein; NLR, neutrophil-to-lymphocyte ratio; LMR, lymphocyte-to-monocyte ratio; PLR, platelet-to lymphocyte ratio; PNI, prognostic nutritional index. Factors which made statistically significant differences are shown in bold.

**Table 3:** Multivariate Cox proportional hazards regression analysis.

Variables	HR	95% CI	p value
**NF2 (+)**	**4.94**	**165-14.78**	**0.0043**
Tumor size (≧30mm)	0.89	0.30-2.70	0.8411
Cyst (+)	2.29	0.83-6.31	0.1093
Removal rate (non-GTR)	3.15	0.69-14.38	0.1380
Adjuvant radiotherapy (+)	1.56	0.42-5.82	0.5045
**NLR (≧2.21)**	**2.82**	**1.04-7.68**	**0.0423**

NF2, neurofibromatosis type 2; GTR, gross total resection; NLR, neutrophil-to-lymphocyte ratio. Factors which made statistically significant differences are shown in bold.

### Subgroup analysis

**Figure 4:**
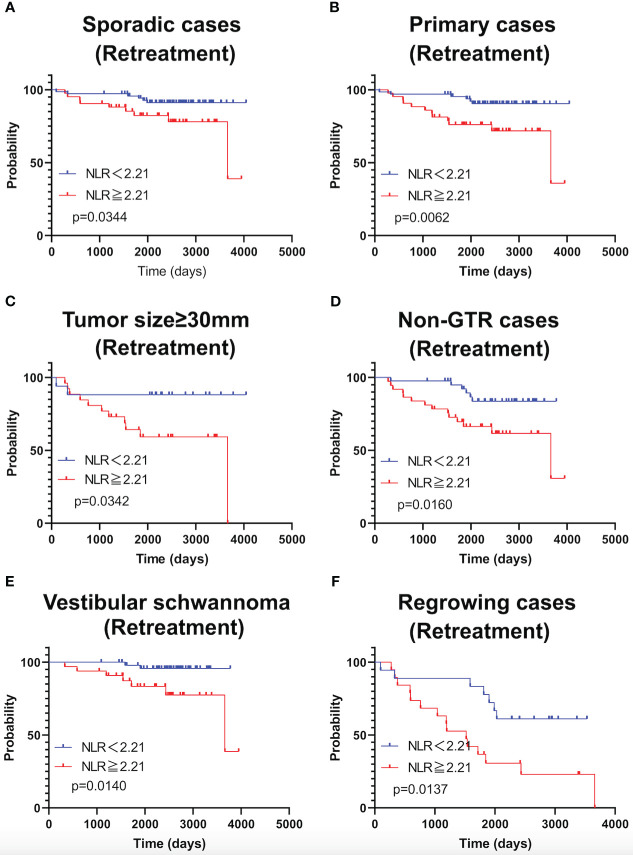
Kaplan-Meier curves of TFS related to sporadic cases **(A)**, primary cases **(B)**, large tumor (≥30mm) **(C)**, Non-GTR cases **(D)**, cases with vestibular schwannomas **(E)**, and growing cases **(F)** are shown.

As shown in **Figure 4**, TFS was significantly shorter in patients with NLR ≥2.21 in the following subgroups: sporadic schwannoma, primary schwannoma, schwannoma ≥30 mm in size, STR, vestibular schwannoma, and postoperative recurrence. Subgroup analysis of NF2 patients was not performed because the sample size was small.

## Discussion

In this study, preoperative NLR was an independent predictor of postoperative schwannoma recurrence that required retreatment. It was also applicable to a subgroup of vestibular schwannomas. Ki-67, S100, p53, microvessel density, and macrophage colony stimulating factor have been previously reported as biomarkers with prognostic value in schwannoma (5, 30–32). Histological inflammation and angiogenesis play a role in growth of sporadic and NF2-related vestibular schwannoma (33). CD163+ tumor-associated macrophages in particular have a supportive effect on schwannoma growth (31, 34–36). However, histopathological biomarkers cannot be evaluated preoperatively and therefore cannot contribute to early surgical decision making. In contrast, NLR can be easily calculated using preoperative blood testing. Although a few previous studies have evaluated serum and radiological prognostic factors associated with inflammatory status, only one has demonstrated that NLR is an important predictor of the natural history in schwannomas (18). However, this study did not examine postoperative growth pattern in surgical cases. Although dynamic positron emission tomography can predict inflammation in schwannomas (37), routine use of such a specialized and expensive imaging modality before surgery is not practical.

Based on the natural history of schwannomas, a subset of schwannomas do not exhibit growth after diagnosis (38–40). Similarly, some schwannomas grow slowly or transiently after surgery, while others grow rapidly and require retreatment. It is important to evaluate continuous tumor growth needing active treatment strategies such as surgery and radiotherapy. TFS, which is survival without the need of treatment for recurrence, may allow us to identify distinct prognostic group of schwannomas. Preoperative identification of those with a shorter TFS would assist surgeons and clinicians with treatment decision making. Our findings suggest that preoperative NLR ≥2.21 is a predictor of shorter TFS after surgery and that STR without adjuvant radiotherapy may be enough for those with a lower NLR. This surgical strategy has a merit in preserving neurological function (8). NLR may be essential in order to provide an evidence-based treatment recommendation for the patient with schwannomas.

This study has several limitations. It was retrospective in design and histopathological analysis was not performed. In addition, the relationship between serum inflammatory parameters and histopathological inflammatory status has not been fully elucidated, and our previous study found no association between NLR and histopathological inflammatory cell infiltration in meningioma (22). Moreover, we did not evaluate NLR after surgery, which may also be a useful predictor of recurrence and retreatment. All the cases were benign schwannomas, therefore the relationship between NLR and malignant schwannoma was not assessed. Future prospective studies are warranted to confirm our findings and investigate further.

## Conclusions

Preoperative NLR ≥2.21 before surgery was significantly associated with retreatment after schwannoma resection. NLR may be a novel predictor of retreatment and assist surgeons in preoperative surgical decision making.

## Data availability statement

The raw data supporting the conclusions of this article will be made available by the authors, without undue reservation.

## Ethics statement

The studies involving human participants were reviewed and approved by Keio University Hospital. Written informed consent to participate in this study was provided by the participants’ legal guardian/next of kin.

## Author contributions

KT and RT conceptualized, designed, and performed the study and wrote the manuscript. YK and KK assisted in the acquisition of data. TA and MT assisted with discussion and review of the manuscript. All authors contributed to the article and approved the submitted version.

## Supplementary material

Supplementary Figure 1 Receiver operating characteristic curves for recurrence and retreatment

Supplementary Figure 2 Kaplan–Meier recurrence-free survival curves with patients stratified according to neurofibromatosis type 2 status and optimal neutrophil-to-lymphocyte ratio cut-off value
