# Supplementary material for: Prognostic significance of preoperative neutrophil-to-lymphocyte ratio in surgically resected schwannomas
Source: Front Oncol. 2023 Feb 10;13:1099384. doi: 10.3389/fonc.2023.1099384 (PMC9950385; doi:10.3389/fonc.2023.1099384)
Supplement: Supplementary file 1 [file Table_1.docx]

**Supplementary Table 1. Patient and tumor characteristics related to recurrence**

NLR, neutrophil-to-lymphocyte ratio; SD, standard deviation; NF2, neurofibromatosis type 2; CN, cranial nerve; GTR, gross total resection

| Clinical feature | All cases | Baseline NLR | | P value |
| --- | --- | --- | --- | --- |
|  |  | <2.03 | ≧2.03 |  |
|  |  | No.(%) | No.(%) |  |
| Patient number | 124 | 59 (47.6) | 65 (52.4) |  |
| Age (means±SD) |  | 50.6±12.7 | 46.6±14.6 | 0.1094 |
| Sex |  |  |  | 0.3665 |
| Male | 55 | 26 (47.3) | 29(52.7) |  |
| Female | 69 | 39 | 30 |  |
| NF2 |  |  |  | 0.1144 |
| + | 11 | 3 (27.3) | 8 (72.7) |  |
| - | 113 | 62 (54.9) | 51 (45.1) |  |
| Tumor status |  |  |  | 0.7841 |
| primary | 109 | 58 (53.2) | 51 (46.8) |  |
| recurrent | 15 | 7 (46.7) | 8 (53.3) |  |
| Tumor origin |  |  |  | 0.3128 |
| CN 8 | 91 | 45 (49.5) | 46 (50.5) |  |
| non CN 8 | 33 | 20 (60.6) | 13 (39.4) |  |
| Neurological symptoms |  |  |  | 0.7817 |
| + | 110 | 57 (51.8) | 53 (48.2) |  |
| - | 14 | 8 (57.1) | 6 (42.9) |  |
| Tumor size (mm) |  | 25.2±13.0 | 27.9±12.3 | 0.2407 |
| Brain compression |  |  |  | 0.7088 |
| + | 79 | 40 (50.6) | 39 (49.4) |  |
| - | 45 | 25 (55.6) | 20 (44.4) |  |
| Tumor cyst |  |  |  | 0.5895 |
| + | 59 | 29 (49.2) | 30 (50.8) |  |
| - | 65 | 36 (55.4) | 29 (44.6) |  |
| Removal rate |  |  |  | 0.0937 |
| GTR | 46 | 29 (63.0) | 17 (37.0) |  |
| non-GTR | 78 | 36 (46.2) | 42 (53.8) |  |
