# Supplementary figures and images for: Prognostic significance of preoperative neutrophil-to-lymphocyte ratio in surgically resected schwannomas
Source: Front Oncol. 2023 Feb 10;13:1099384. doi: 10.3389/fonc.2023.1099384 (PMC9950385; doi:10.3389/fonc.2023.1099384)

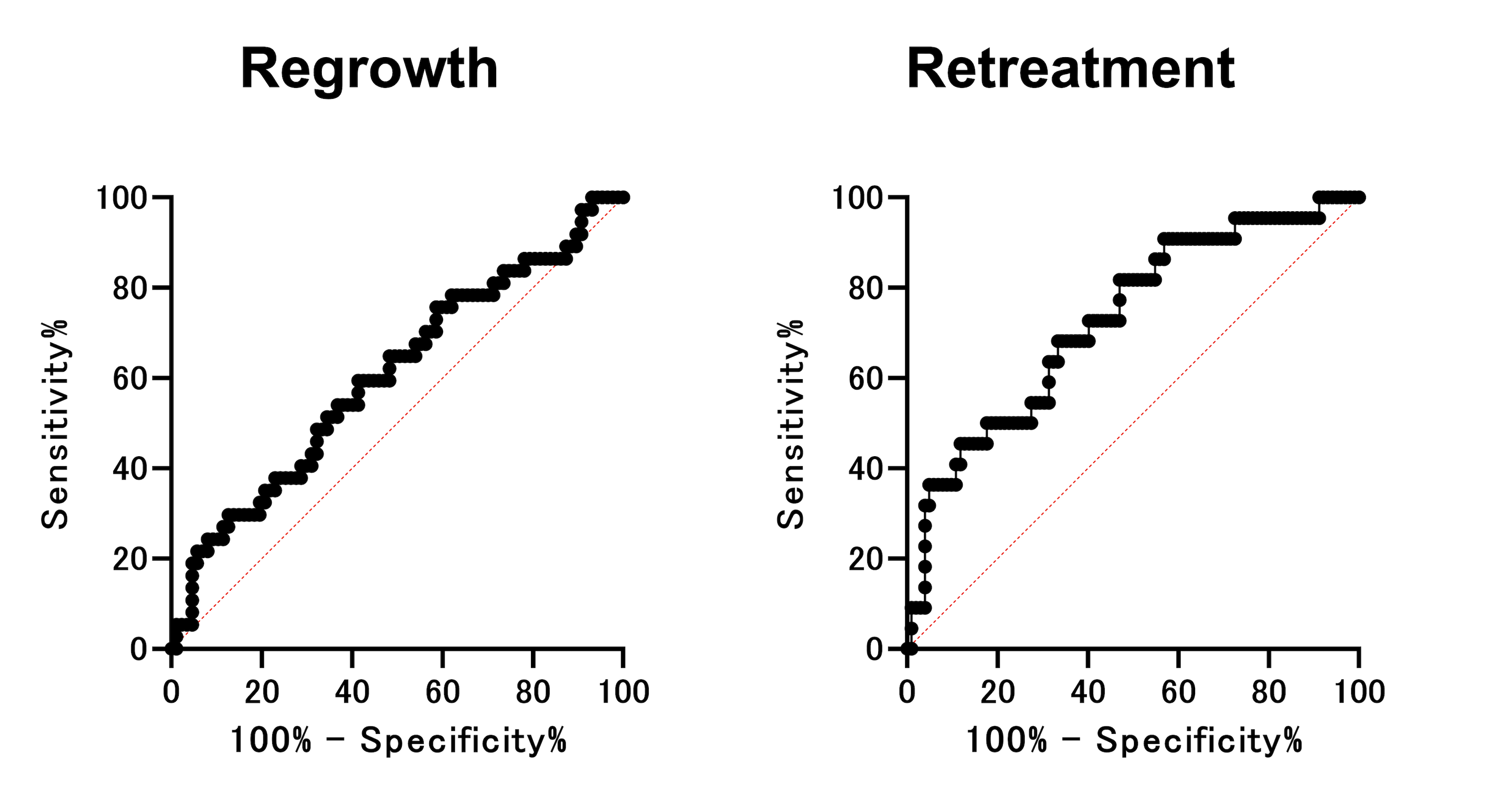

Supplement: Supplementary Figure 1 — Receiver operating characteristic curves for recurrence and retreatment [file Image_1.png]

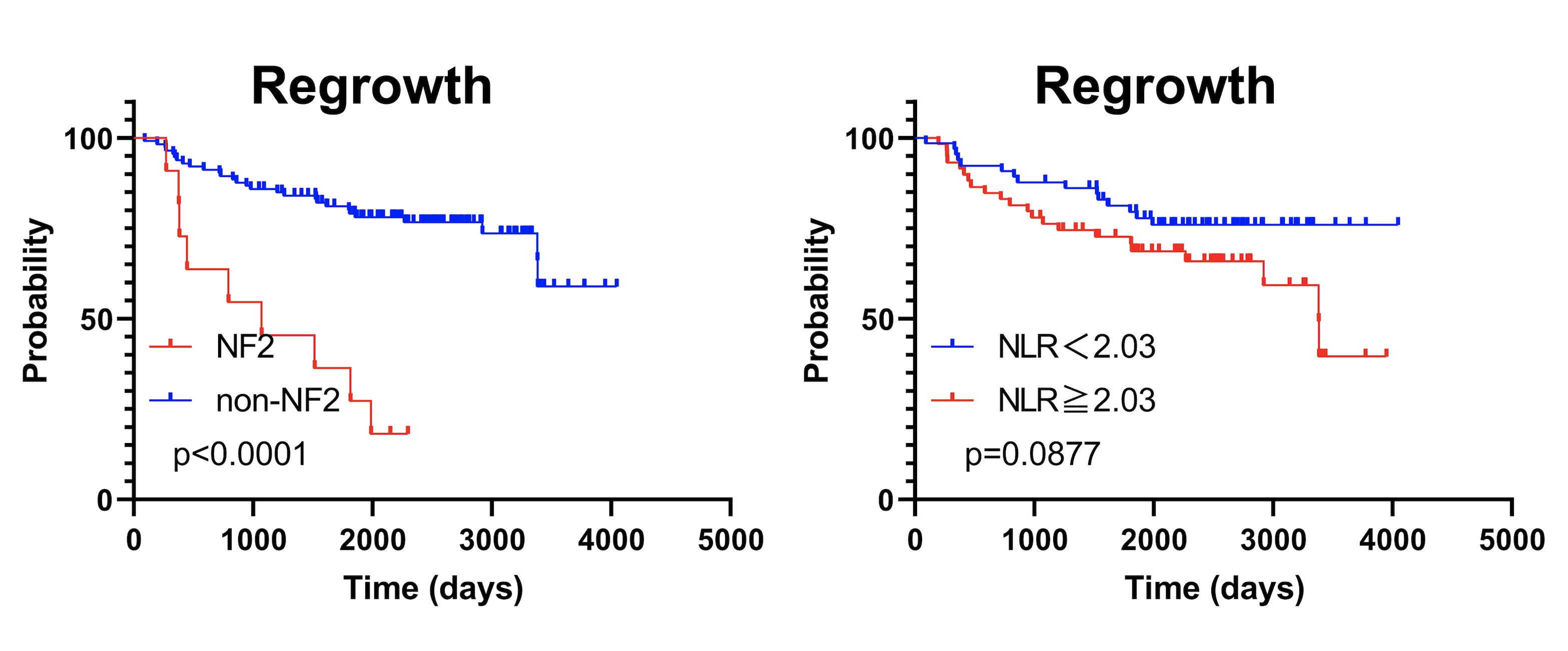

Supplement: Supplementary Figure 2 — Kaplan–Meier recurrence-free survival curves with patients stratified according to neurofibromatosis type 2 status and optimal neutrophil-to-lymphocyte ratio cut-off value [file Image_2.png]
